# Photocatalytic Hydrogen Evolution Activity of Nitrogen/Fluorine-Codoped Rutile TiO_2_

**DOI:** 10.1021/acsomega.3c06492

**Published:** 2023-10-23

**Authors:** Akinobu Miyoshi, Megumi Okazaki, Kosaku Kato, Tomoki Kanazawa, Toshiyuki Yokoi, Shunta Nishioka, Shunsuke Nozawa, Akira Yamakata, Kazuhiko Maeda

**Affiliations:** † Department of Chemistry, School of Science, Tokyo Institute of Technology, 2-12-1-NE-2 Ookayama, Meguro-ku, Tokyo 152-8550, Japan; ‡ Graduate School of Natural Science and Technology, Okayama University, 3-1-1 Tsushima-naka, Kita-ku, Okayama 700-8530, Japan; § Institute of Materials Structure Science, High Energy Accelerator Research Organization, 1-1 Oho, Tsukuba, Ibaraki 305-0801, Japan; ∥ Nanospace Catalysis Unit, Institute of Innovative Research, Tokyo Institute of Technology, 4259 Nagatsuta-cho, Midori-ku, Yokohama 226-8503, Japan; ⊥ Living Systems Materialogy (LiSM) Research Group, International Research Frontiers Initiative (IRFI), Tokyo Institute of Technology, 4259 Nagatsuta-cho, Midori-ku, Yokohama, Kanagawa 226-8502, Japan

## Abstract

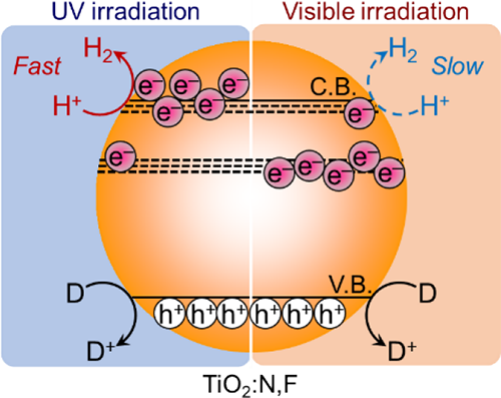

The development of a photocatalyst capable of evolving H_2_ from water under visible light is important. Here, the photocatalytic activity of N/F-codoped rutile TiO_2_ (TiO_2_:N,F) for H_2_ evolution was examined with respect to metal cocatalyst loading and irradiation conditions. Among the metal species examined, Pd was the best-performing cocatalyst for TiO_2_:N,F under UV–vis irradiation (λ > 350 nm), producing H_2_ from an aqueous methanol solution. The H_2_ evolution activity was also dependent on the state of the loaded Pd species on the TiO_2_:N,F, which varied depending on the preparation conditions. Pd/TiO_2_:N,F prepared by an impregnation–H_2_ reduction method, showed the highest performance. However, the activity of the optimized Pd/TiO_2_:N,F toward H_2_ evolution from an aqueous methanol solution was negligibly small under visible-light irradiation (λ > 400 nm), although the use of an ethylenediaminetetraacetic acid disodium salt as an electron donor resulted in observable H_2_ evolution. Transient absorption spectroscopy revealed that although a relatively large population of reactive electrons was generated in the TiO_2_:N,F under 355 nm UV-pulse photoexcitation, the density of reactive electrons generated under 480 nm visible light was lower. This wavelength-dependent behavior in photogenerated charge carrier dynamics could explain the different photocatalytic activities of the TiO_2_:N,F catalysts under different irradiation conditions.

## Introduction

Photocatalytic water splitting is attracting attention as a possible means to convert solar energy into a chemical energy carrier such as H_2_.^1−7^ Although metal oxide photocatalysts with high quantum efficiencies have been developed, most of them can absorb only UV light because of their wide band gap.^2,8,9^ UV light accounts for only ∼5% of the solar spectrum; therefore, utilization of visible light is necessary to achieve greater solar energy conversion efficiency.

In this regard, narrow-bandgap mixed-anion compounds are attractive because they can absorb visible light.^10−13^ N doping is a conventional method to render wide-band-gap metal oxides with the ability to absorb visible light by narrowing their energy gap, as studied both experimentally and theoretically.^14−16^ The valence-band maxima of N-doped oxides are mainly composed of N 2p states, which are located just above the O 2p states, enabling the realization of band gaps narrower than those of the corresponding metal oxides.

Given the high Earth abundance of TiO_2_, development of a TiO_2_-based photocatalyst that exhibits a visible-light response might be ideal in terms of its potential for use in large-scale applications.^4,17^ Recently, we developed N/F-codoped rutile TiO_2_ (TiO_2_:N,F), which functions as an O_2_-evolution photocatalyst in visible-light Z-scheme water-splitting systems in combination with H_2_-evolving Ru-loaded, Rh-doped SrTiO_3_ (Ru/SrTiO_3_:Rh) and [Co(bpy)_3_]^3+/2+^ (bpy = 2,2′-bipyridyl) as a mediator.^18,19^ When TiO_2_:N,F nanoparticles were used instead of bulk TiO_2_:N,F, the water splitting rate for the Z-scheme tripled. Although oxide-based mixed-anion compounds that contain less-electronegative anions are inherently unstable toward photooxidation reactions,^1,8,12^ such N-doped TiO_2_ analogs have demonstrated high stability during water oxidation to O_2_.^18,20,21^

In addition to the development of such an O_2_-evolution photocatalyst, the development of a new H_2_-evolution photocatalyst for visible-light-driven Z-scheme water splitting systems is strongly demanded.^2,7^ In particular, the use of Earth-abundant elements for photocatalysts is desirable from a sustainability viewpoint. Anatase TiO_2_:N,F has been reported to exhibit H_2_-evolution activity from an aqueous methanol solution under visible light.^22−24^ Although the rutile TiO_2_:N,F photocatalyst has the ability to thermodynamically reduce H_2_O (or H^+^) into H_2_,^18^ its water reduction activity has not been investigated in detail.

In the present study, we optimized the loading of cocatalysts on rutile TiO_2_:N,F. The loading of Pd, Pt, or Ir was found to enhance H_2_ evolution under sacrificial conditions. Also, the catalyst loaded with Pd via the impregnation–H_2_ reduction method [(Imp–H_2_)Pd/TiO_2_:N,F] showed greater activity than the catalyst loaded with Pd via the photodeposition (PD) method [(PD)Pd/TiO_2_:N,F], demonstrating a H_2_ evolution rate of 55 μmol h^–1^ under irradiation with λ > 350 nm light. However, the photocatalytic activity of Pd- or Pt-loaded TiO_2_:N,F under visible light was very low. The different activities with respect to the photoexcitation conditions were investigated by transient absorption spectroscopy.

## Experimental Section

### Materials and Reagents

Rutile TiO_2_ (JRC-TIO-16), (NH_4_)_2_TiF_6_ (Wako Pure Chemicals, 95.0%), Na_2_PdCl_4_ (Wako Pure Chemicals, 95.0%), PdO (Wako Pure Chemicals, 98.0%), H_2_PtCl_4_ (Wako Pure Chemicals, 98.5%), Na_2_IrCl_6_·6H_2_O (Kanto Chemicals, 97.0%), HAuCl_4_ (Wako Pure Chemicals, 99.0%), Na_3_RhCl_6_·6H_2_O (Mitsuwa Chemical), RuCl_3_·*n*H_2_O (Furuya Metal), and EDTA·2Na (Dojindo Laboratories, 99.5%) were used as received without further purification.

### General Characterization

The materials were characterized by powder X-ray diffraction (XRD; Rigaku MiniFlex 600; Cu Kα), UV–vis diffuse-reflectance spectroscopy (DRS; JASCO, V-670), and scanning electron microscopy (SEM; Hitachi High-Technologies, SU9000). X-ray absorption fine structure (XAFS) measurements were performed at beamline NW10A (PF-AR) of the High Energy Accelerator Research Organization, Tsukuba, Japan. The X-ray energy was varied by using a Si(111) double-crystal monochromator. The data were processed using Athena.^25^

### Preparation of TiO_2_:N,F

Rutile TiO_2_:N,F was synthesized according to the previously reported method by mixing rutile TiO_2_ (JRC-TIO-16) and (NH_4_)_2_TiF_6_ in a molar ratio of 95:5 using an agate mortar and pestle.^18,19^ The mixture was loaded onto a Ni plate to prevent contamination from Al_2_O_3_ and placed at the center of an alumina tube reactor.^22^ After the system was purged with dry NH_3_, the reactor was heated to 673 K (ramp: 10 K min^–1^) for 15 h under dry NH_3_ flow (flow rate: 100 mL min^–1^). The specific surface area of the material thus obtained was 40 m^2^ g^–1^, as determined by an N_2_-adsorption measurement at 77 K.

### Impregnation of Metal Species onto TiO_2_:N,F

TiO_2_:N,F was dispersed into water containing metal species (1 wt % metal relative to TiO_2_:N,F). As the metal precursors, Na_2_PdCl_4_, H_2_PtCl_6_, Na_2_IrCl_6_·6H_2_O, HAuCl_4_, Na_2_RhCl_6_·6H_2_O, or RuCl_3_·*n*H_2_O was used. The powder suspension was heated on a steam bath while being continuously stirred with a glass rod until dry. The dried powder was lightly ground using an agate mortar and pestle and then heated at 473 K for 1 h under a H_2_ flow (20 mL min^–1^) or in air.

### Photocatalytic Reactions

Photocatalytic reactions were conducted in a Pyrex top-irradiation-type reaction vessel connected to a closed gas circulation system.^26^ In a typical H_2_ evolution reaction, TiO_2_:N,F was dispersed in 140 mL of 10 vol % methanol aqueous solution or 10 mM EDTA·2Na solution, where methanol or EDTA acted as an electron donor. For in situ PD experiments, an appropriate metal precursor (1 wt % metal relative to TiO_2_:N,F) was dissolved in the solution. The system was evacuated several times to remove air and then a small amount of Ar gas was introduced prior to irradiation. For the light source, a 300 W Xe lamp (Cermax, PE300BF) operating with an output current of 20 A or a solar simulator (Asahi Spectra HAL-320, irradiation area: 16 cm^2^) was used. Light from the Xe lamp was passed through a water filter in combination with a CM-1 cold mirror. To change the irradiation wavelength, an L42 cutoff filter was used. Evolved gases were analyzed by an online gas chromatograph (Shimadzu, GC-2014s equipped with a thermal conductivity detector and MS-5A column; Ar carrier gas). The solution was kept at room temperature with a flow of cooling water during the reaction. The material after the reaction was collected by filtration and dried at 343 K for further characterization.

### Transient Absorption Spectroscopy

Transient absorption spectroscopy measurements were conducted using a custom-made spectrometer.^27^ TiO_2_:N,F samples were fixed onto a CaF_2_ plate at a density of 1.5 mg cm^–2^ and placed in an IR cell for measurement. The samples were photoexcited using 355 nm pulses from a Nd:YAG laser (Continuum Surelite I with Surelite OPO; duration, 6 ns; power, 2 mJ; repetition rate, 5–0.1 Hz), and transient absorption in the visible to mid-IR region was measured with the sample under a N_2_ atmosphere. The same measurements were also conducted by using 480 nm pulses (power, 5 mJ). The time resolution of this spectrometer was limited to 1–2 μs by the bandwidth of the amplifier (Stanford Research Systems, SR560, 1 MHz).

## Results and Discussion

### PD of Metal Species onto Rutile TiO_2_:N,F

**Figure 1:**
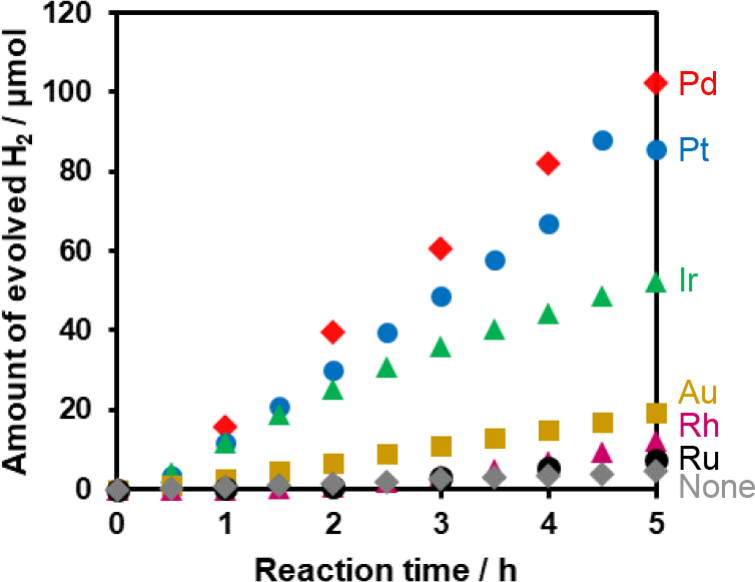
Time courses of H_2_ evolution from TiO_2_:N,F dispersed in aqueous methanol solutions containing various metal precursors under UV–vis (λ > 350 nm) irradiation. Reaction conditions: TiO_2_:N,F, 150 mg; metal precursor (Na_2_PdCl_4_, H_2_PtCl_6_, Na_2_IrCl_6_·6H_2_O, HAuCl_4_, Na_2_RhCl_6_·6H_2_O, or RuCl_3_·*n*H_2_O), 1 wt % (metal content relative to TiO_2_:N,F); 10 vol % aqueous methanol solution, 140 mL; light source, 300 W Xe lamp fitted with a CM-1 mirror.

As the first step, the PD method, which does not require a heating procedure,^28^ was used as a cocatalyst loading method. TiO_2_:N,F nanoparticles, which showed greater water oxidation activity than bulk TiO_2_:N,F,^19^ was used unless otherwise stated. Figure 1 shows the time courses of H_2_ evolution from a 10 vol % aqueous methanol solution containing various metal precursors under irradiation with λ > 350 nm light. Irrespective of the metal species, an induction period was observed at the beginning, suggesting *in situ* PD of the metal species onto the TiO_2_:N,F. The higher H_2_ evolution rate for the metal precursor-containing reaction solution than for the solution without a metal (indicated as “none”) suggests that the *in situ* loaded metal species functioned as cocatalysts for H_2_ evolution. Among the investigated metals, Pd exhibited the highest activity, with the activity of Pt being slightly lower. Although Ir initially showed activity comparable to that of Pd and Pt, its H_2_ evolution rate decreased after 1 h. These results suggest that the Ir state changed during the reaction and/or that the loading of excess Ir following the initial formation of active Ir species resulted in deactivation.

### Comparison of Impregnation and PD Methods for H_2_ Evolution Reaction under UV–Vis Irradiation

**Figure 2:**
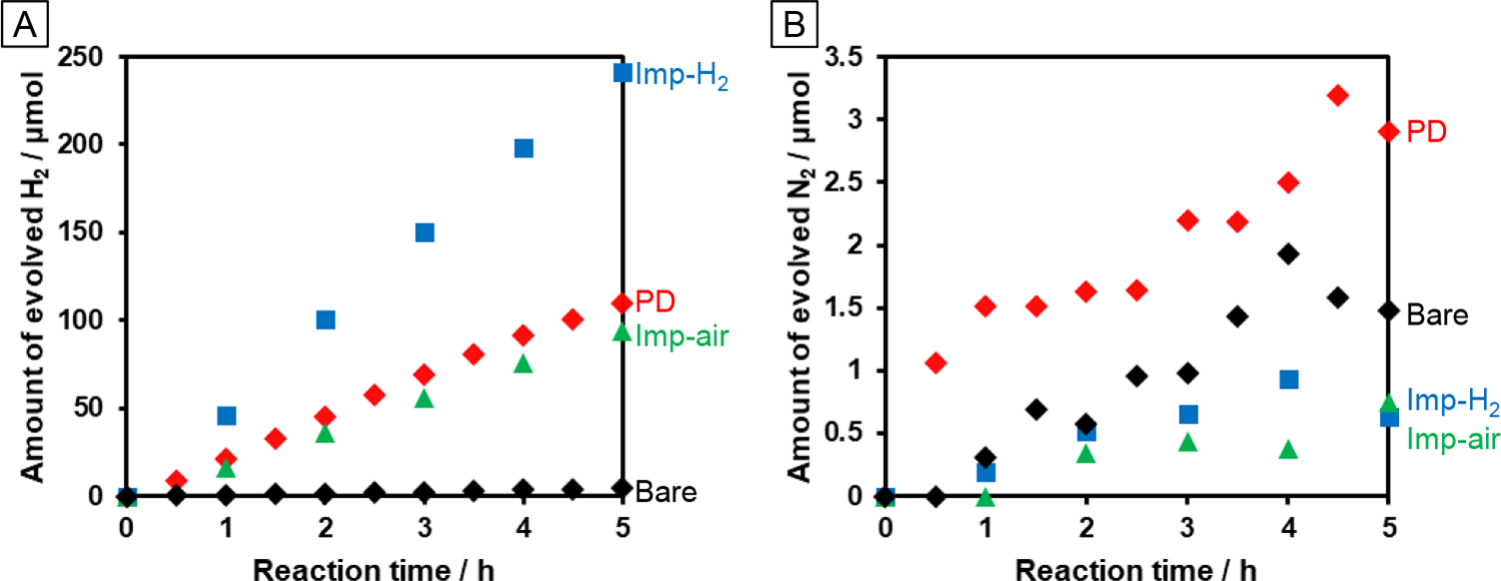
Time courses of (A) H_2_ and (B) N_2_ evolution from Pd(1 wt %)/TiO_2_:N,F catalysts prepared using different loading methods when the catalysts were dispersed in 10 vol % aqueous methanol and irradiated with UV–Vis (λ > 350 nm) light. Reaction conditions: catalyst, 50 mg; 10 vol % aqueous methanol solution, 140 mL; light source, 300 W Xe lamp fitted with a CM-1 mirror. For *in situ* photodeposition, Na_2_PdCl_4_ (1 wt %) was added to the reaction solution.

The Pd cocatalyst loading methods were optimized under sacrificial conditions by using methanol as an electron donor. Figure 2A shows the time courses of H_2_ evolution from Pd/TiO_2_:N,F prepared by *in situ* PD [(PD)Pd/TiO_2_:N,F], impregnation followed by H_2_ reduction [(Imp–H_2_)Pd/TiO_2_:N,F], and impregnation followed by heating in air [(Imp–air)Pd/TiO_2_:N,F]. The catalyst prepared by Imp–H_2_ showed twofold greater activity than that prepared by PD. All of the Pd/TiO_2_:N,F catalysts showed a ∼10 min induction period, suggesting that the catalyst state changed before the start of H_2_ evolution. In the case of (PD)Pd/TiO_2_:N,F, the *in situ* formation of Pd species on the surface of TiO_2_:N,F might have caused such an induction period. However, both the (Imp–H_2_)Pd/TiO_2_:N,F and (Imp–air)Pd/TiO_2_:N,F catalysts showed a similar induction period. These results suggest that the state of the TiO_2_:N,F and/or loaded Pd also changed during the initial period. A change in the TiO_2_:N,F state during light irradiation is also supported by the observation of a ∼40 min induction period for H_2_ evolution from the bare TiO_2_:N,F catalyst (Figure S1). In addition, an increase in absorption at ∼450 nm, which is caused by N doping, was detected in the DRS of the catalyst after the reaction (Figure S2A). Because the XRD pattern of the catalyst (Figure S2B) did not change after the reaction, the change in the DRS likely resulted from changes that occurred in limited regions, such as the surface. Notably, all of the Pd/TiO_2_:N,F catalysts showed a shorter induction period than the bare TiO_2_:N,F. If this induction period represents the time required to accumulate sufficient carriers in the semiconductor, then the shorter induction period for the Pd/TiO_2_:N,F catalysts might indicate a decrease in the overpotential needed to drive H_2_ evolution as a consequence of the loaded cocatalysts. The (Imp–H_2_)Pd/TiO_2_:N,F catalyst showed enhanced absorption at λ > 500 nm, whereas the (Imp–air)Pd/TiO_2_:N,F catalyst did not. The enhanced absorption in this region is likely the result of reduced Ti species generated during the H_2_ reduction process.^29^

The effect of the cocatalyst loading is also apparent in the time course of N_2_ evolution (Figure 2B), which results from self-oxidation of TiO_2_:N,F.^22^ Compared with a bare sample, the metal-photodeposited TiO_2_:N,F (PD) catalyst showed enhanced N_2_ evolution. This result suggests that the loaded Pd species enhanced H_2_ evolution and that the increase in the number of holes left in the semiconductor drove the self-oxidation reaction. However, the catalysts prepared by Imp–H_2_ and Imp–air showed suppressed N_2_ evolution, suggesting that the heat treatment resulted in improved stability of the doped nitrides or the removal of weakly bonded nitrogen species from the surface region of the catalysts.

**Figure 3:**
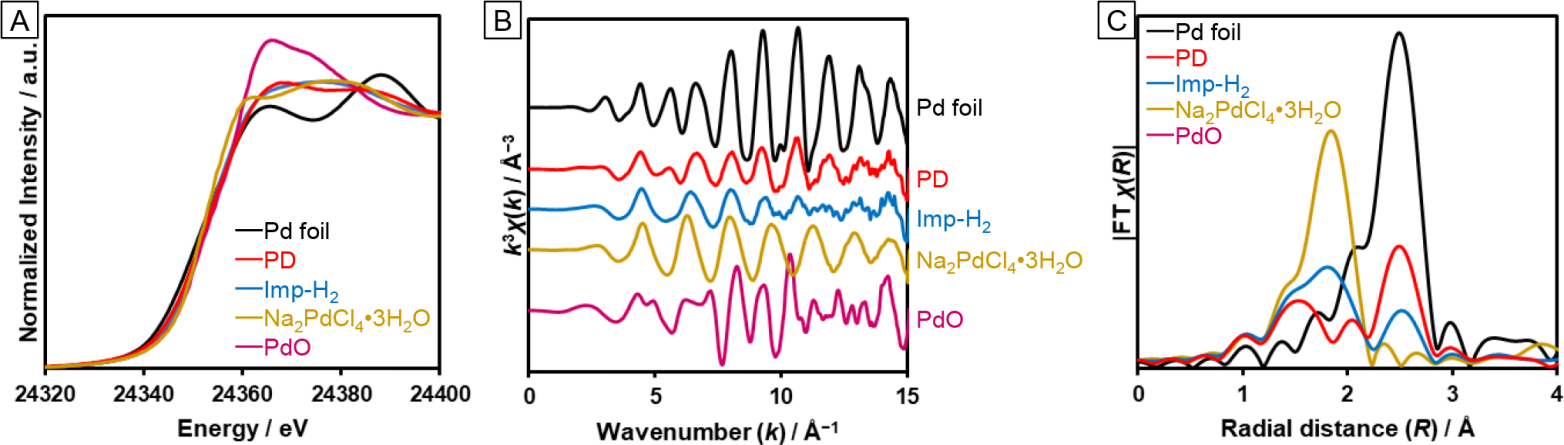
Pd K-edge (A) XANES spectra, (B) EXAFS spectra, and (C) Fourier transformed EXAFS spectra of as-prepared Pd(1 wt %)/TiO_2_:N,F catalysts. Fourier transformation range: 3–13 Å^–1^.

To characterize the loaded Pd species, we conducted Pd K-edge XAFS measurements for the catalysts prepared by Imp–H_2_ and PD. The X-ray absorption near edge structure (XANES) spectra (Figure 3A) suggest that the Pd species deposited on TiO_2_:N,F was a mixture of metallic Pd and Na_2_PdCl_4_. Oscillations in the extended X-ray absorption fine structure (EXAFS) region (Figure 3B) support this finding. The shapes of the XANES and EXAFS oscillations indicate that the catalyst prepared by PD contains more Pd metal than that prepared by Imp–H_2_. Fourier transformed EXAFS spectra are shown in Figure 3C. The disappearance of the peak at 1.9 Å in the spectrum of the catalyst prepared by PD indicates the (partial) decomposition of Na_2_PdCl_4_ during the PD procedure.

**Figure 4:**
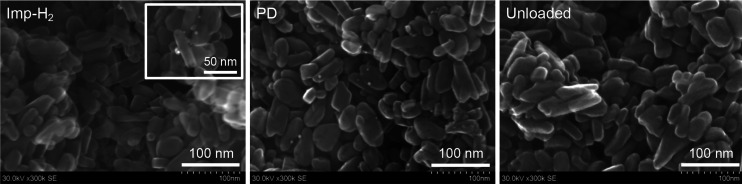
SEM images of Pd-loaded TiO_2_:N,F samples and a bare TiO_2_:N,F sample. The inset in the left panel is a magnified image of a selected area.

The scanning electron microscopy (SEM) images in Figure 4 reveal that ∼5 nm Pd nanoparticles were deposited onto the surface of TiO_2_:N,F in the photodeposited catalyst. By contrast, few deposits were observed for the (Imp–H_2_)Pd/TiO_2_:N,F catalyst, although careful observation in a selected area revealed a few ∼5 nm Pd species (Figure 4 inset). Thus, not only the valence state of Pd but also the morphological character were found to differ among the prepared catalysts.

These results indicate that the enhanced activity of the (Imp–H_2_)Pd/TiO_2_:N,F catalyst compared with that of the (PD)Pd/TiO_2_:N,F or (Imp–air)Pd/TiO_2_:N,F catalyst might be caused by differences in TiO_2_:N,F itself and in the loaded Pd species. The DRS results suggest that the (Imp–H_2_)Pd/TiO_2_:N,F had a greater donor (electron) concentration (Figure S2A). An increase in the electron concentration with appropriate donor doping has been reported to not only enhance charge separation and electron conductivity but also prolong the lifetime of free electrons.^30−32^ Thus, partial reduction of Ti in TiO_2_:N,F might be one of the reasons for the enhanced activity. However, the state of the loaded Pd was revealed to differ depending on the loading method. Although both the (Imp–H_2_)Pd/TiO_2_:N,F and (PD)Pd/TiO_2_:N,F samples appear to contain both metal and chloride species, the (Imp–H_2_)Pd/TiO_2_:N,F sample has a higher chloride fraction than the (PD)Pd/TiO_2_:N,F sample. This result suggests that the remaining chloride species might be responsible for the high activity of the (Imp–H_2_)Pd/TiO_2_:N,F sample.

The chloride species can enhance activity through two possible mechanisms: (1) the chloride itself can act as an electron donor or (2) the chloride can function as a precursor for the “real” catalyst.^33,34^ Possibility (1) is less likely than possibility (2) because no H_2_ evolution was observed in the absence of methanol as an electron donor. SEM observations showed that highly dispersed Pd species, enabled by the Imp–H_2_ procedure, on TiO_2_:N,F likely contribute, at least in part, to the higher photocatalytic activity of the (Imp–H_2_)Pd/TiO_2_:N,F catalyst because smaller nanoparticle catalysts are generally beneficial to photocatalytic H_2_ evolution.^5,8,35^

### Photocatalytic H_2_ Evolution Activity of M/TiO_2_:N,F under Visible-Light Irradiation

In the previous section, Pd was shown to be the most effective H_2_ evolution cocatalyst for TiO_2_:N,F in aqueous methanol under UV–vis irradiation. We subsequently conducted H_2_ evolution reactions using Pd/TiO_2_:N,F under visible-light irradiation (λ > 400 nm). However, no H_2_ evolution was observed from aqueous methanol (Table 1). The activity was also negligible when Pt/TiO_2_:N,F was used.

**Table 1:** H_2_ Evolution Rates for Cocatalyst-Loaded TiO_2_:N,F (Loading Amount 1 wt % via the Imp–H_2_ Method) from Electron-Donor Solutions^a^

cocatalyst	electron donor	irradiation wavelength	H_2_ evolution rate (μmol h^–1^)
Pd	MeOH	>350 nm^b^	55
Pd	MeOH	>400 nm^c^	N.D.
Pt	MeOH	>400 nm^c^	N.D.
Pd	EDTA•2Na	>400 nm^c^	1.7

a Reaction conditions: catalyst, 50 mg; 10 vol% methanol or 10 mM EDTA·2Na aqueous solution 140 mL; light source.

b 300 W Xe lamp fitted with a CM-1 mirror.

c 300 W Xe lamp fitted with a CM-1 mirror and a L42 cutoff filter.

**Figure 5:**
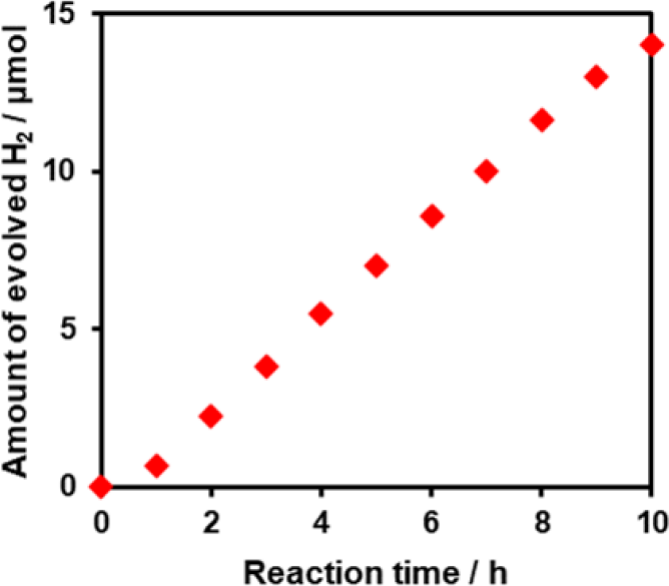
Time course of H_2_ evolution from (Imp–H_2_)Pd/TiO_2_:N,F under visible-light irradiation (λ > 400 nm) in the presence of EDTA·2Na. Reaction conditions: Pd/TiO_2_:N,F, 50 mg; 10 mM EDTA·2Na aqueous solution, 140 mL; light source, 300 W Xe lamp fitted with a CM-1 mirror and an L42 cutoff filter.

Previous studies involving oxynitride photocatalysts sometimes suggested that methanol was not the optimal electron donor for driving H_2_ evolution.^36^ We therefore investigated other electron donors. H_2_ evolution was observed when the reaction was conducted in an aqueous solution containing dissolved ethylenediaminetetraacetic acid disodium salt (EDTA·2Na), which is a stronger electron donor than methanol. As shown in Figure 5, H_2_ evolution occurred almost linearly at a maximum rate of 1.7 μmol h^–1^. The short induction period observed at the beginning might be a result of photoreduction of the palladium chloride precursors remaining on the powder after H_2_ reduction (Figure 3). When the same reaction was conducted under AM 1.5G simulated sunlight, the maximum rate of H_2_ evolution increased to 7.0 μmol h^–1^ (Figure S3). This increase in activity under weak simulated sunlight compared with that under a high-intensity Xe lamp suggests the importance of the UV component of irradiated light for efficient H_2_ evolution.

### Photogenerated Charge Carrier Dynamics of TiO_2_:N,F

**Figure 6:**
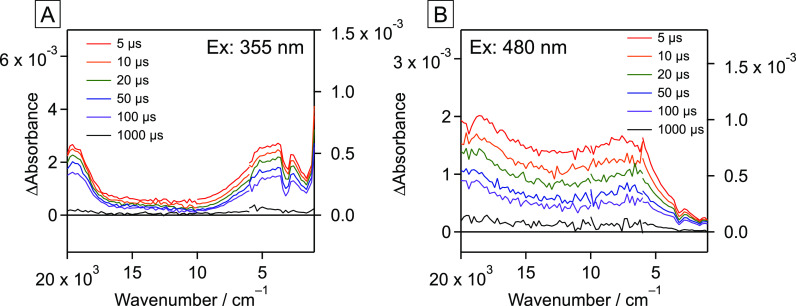
Transient absorption spectra of rutile TiO_2_:N,F: (A) 355 and (B) 480 nm excitation. The spectra were recorded under a N_2_ atmosphere. Transmittance and reflectance were measured below and above 6000 cm^–1^, respectively.

To investigate the reason for the dependence of the H_2_ evolution activity of TiO_2_:N,F on the irradiation wavelength, we investigated the dependence of the carrier dynamics on the excitation wavelength by using transient absorption spectroscopy. Figure 6 shows transient absorption spectra of bare TiO_2_:N,F excited at different laser-pulse wavelengths. The signal at ∼19,500 cm^–1^ is usually ascribed to trapped photogenerated holes.^19,37^ Signals in the range 17,000–2000 cm^–1^ are usually ascribed to deeply trapped electrons, and signals at <2000 cm^–1^, which increase in intensity with decreasing wavenumber, are ascribed to free or shallowly trapped electrons.^37,38^ The negative peak at 3200 cm^–1^ is due to the noise caused by adsorbed water. Notably, the spectral shape in Figure 6A differs from that recorded under 480 nm irradiation (Figure 6B). This difference indicates that the carrier dynamics in TiO_2_:N,F are excitation-wavelength-dependent, which might be the cause of the difference in the photocatalytic activities of the TiO_2_:N,F catalysts under different irradiation conditions.

Notably, under 480 nm excitation, the absorption for deeply trapped electrons shifted to higher wavenumbers compared to that under 355 nm excitation. Although the reason for this difference is not yet clear, the number of free or shallowly trapped electrons appears to be associated with the dependence of the H_2_ evolution activity on the excitation wavelength. That is, a high H_2_ evolution rate under UV irradiation might be associated with a higher concentration of free or shallowly trapped electrons, whereas the absence of H_2_ evolution under visible-light irradiation might be associated with a lower concentration of free or shallowly trapped electrons as a result of the fast deep trapping of electrons.

## Conclusions

This work investigated the photocatalytic activity of rutile-type TiO_2_:N,F for H_2_ evolution with respect to the metal cocatalyst loading and irradiation wavelength. Among the samples examined, (Imp–H_2_)Pd/TiO_2_:N,F demonstrated the highest rate of H_2_ evolution from aqueous methanol under UV- and visible-light irradiation (λ > 350 nm). Under visible-light irradiation (λ > 420 nm), however, the H_2_ evolution rate was substantially lower. This result was attributed to the lower population of reactive electrons generated under visible light in TiO_2_:N,F as a result of the fast deep trapping of excited electrons.

## Supporting Information Available

Time course of H_2_ evolution using unmodified TiO_2_:N,F under UV–vis (λ > 350 nm) irradiation; UV–vis DRS and XRD patterns for Pd/TiO_2_:N,F before and after the reaction; and time course of H_2_ evolution from Pd (Imp-H_2_)/TiO_2_:N,F under simulated sunlight in the presence of EDTA·2Na (PDF)
