# Supplementary material for: Photocatalytic Hydrogen Evolution Activity of Nitrogen/Fluorine-Codoped Rutile TiO2
Source: ACS Omega. 2023 Oct 23;8(44):41809–15. doi: 10.1021/acsomega.3c06492 (PMC10634042; doi:10.1021/acsomega.3c06492)
Supplement: Supplementary file 1 — ao3c06492_si_001.pdf [file ao3c06492_si_001.pdf]

Supporting Information (SI)

Photocatalytic Hydrogen Evolution Activity of Nitrogen/Fluorine-Codoped Rutile TiO_2

*Akinobu Miyoshi,[1] Megumi Okazaki,[1] Kosaku Kato,[2] Tomoki Kanazawa,[3] Toshiyuki Yokoi,[4] Shunta Nishioka,[1] Shunsuke Nozawa,[3] Akira Yamakata,[2] Kazuhiko Maeda,[1,5]**

[1] Department of Chemistry, School of Science, Tokyo Institute of Technology, 2-12-1-NE-2 Ookayama, Meguro-ku, Tokyo 152-8550, Japan.

[2] Graduate School of Natural Science and Technology, Okayama University, 3-1-1 Tsushima-naka, Kita-ku, Okayama, Japan.

[3] Institute of Materials Structure Science, High Energy Accelerator Research Organization, 1-1 Oho, Tsukuba, Ibaraki 305-0801, Japan.

[4] Nanospace Catalysis Unit, Institute of Innovative Research, Tokyo Institute of Technology, 4259 Nagatsuta-cho, Midori-ku, Yokohama 226-8503, Japan.

[5] Living Systems Materialogy (LiSM) Research Group, International Research Frontiers Initiative (IRFI), Tokyo Institute of Technology, 4259 Nagatsuta-cho, Midori-ku, Yokohama, Kanagawa 226-8502, Japan.

*To whom correspondence should be addressed; Email: maedak@chem.titech.ac.jp

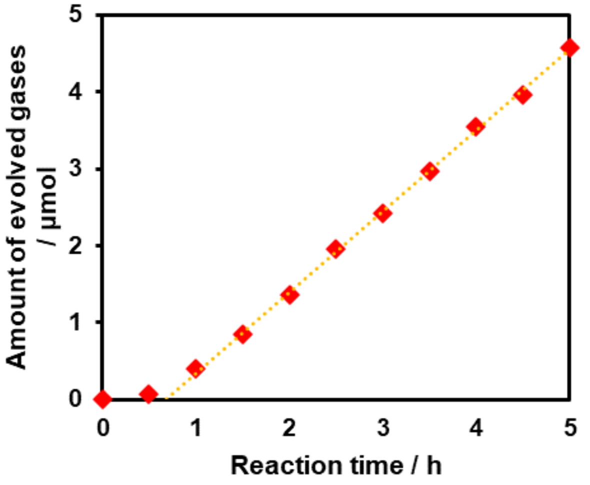

Figure S1. Time course of H_2 evolution using unmodified TiO_2:N,F under UV–Vis ($\lambda > 350$ nm) irradiation. Reaction conditions: TiO_2:N,F, 50 mg; 10 vol.% methanol aqueous solution, 140 mL; light source, 300 W Xe lamp fitted with a CM-1 mirror.

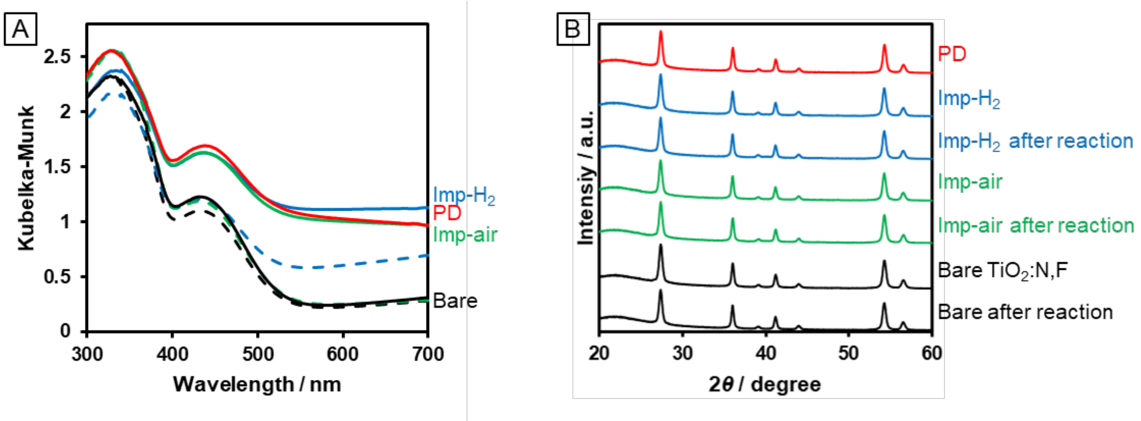

Figure S2. (A) UV–Vis diffuse-reflectance spectra and (B) XRD patterns for Pd/TiO_2:N,F before and after the reaction shown in Figure 2. The broken lines and the solid lines in panel (A) show diffuse-reflectance spectra of the materials before and after reaction, respectively.

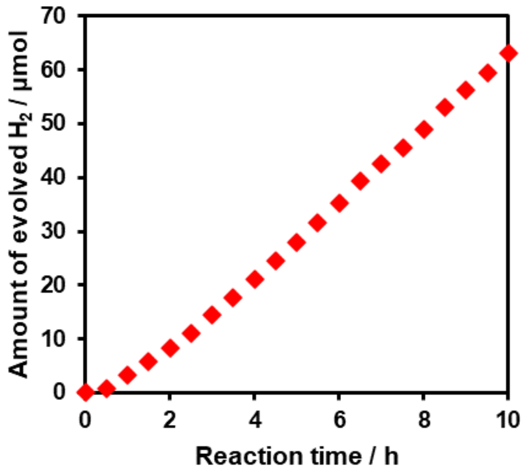

Figure S3. Time course of H_2 evolution from Pd (Imp-H_2)/TiO_2:N,F under simulated sunlight in the presence of EDTA·2Na. Reaction conditions: Pd/TiO_2:N,F, 50 mg; 10 mM EDTA·2Na aqueous solution, 140 mL; light source, a solar simulator (100 mW cm^{-2}).
